# Amino Acids as Sustainable Alternatives to Allergenic Sulfur Vulcanization Accelerators for Carbon Black-Filled Nitrile Rubber

**DOI:** 10.3390/molecules31183250

**Published:** 2026-09-14

**Authors:** Anna Sowińska-Baranowska, Magdalena Maciejewska

**Affiliations:** Department of Chemistry, Institute of Polymer and Dye Technology, Lodz University of Technology, Stefanowskiego Street 16, 90-537 Lodz, Poland

**Keywords:** amino acids, accelerators, crosslinking, vulcanization, carbon black, acrylonitrile–butadiene rubber, properties, biocomposites

## Abstract

Acrylonitrile–butadiene rubber (NBR) is one of the most widely used elastomers in healthcare products owing to its excellent mechanical performance, chemical resistance, and durability. However, conventional sulfur vulcanization systems rely on accelerators such as tetramethylthiuram disulfide (TMTD), 2-mercaptobenzothiazole (MBT), and 1,3-diphenylguanidine (DPG), which are associated with allergenic reactions and potential toxicological concerns. The replacement of these substances with naturally derived compounds represents an attractive strategy for developing safer and more sustainable elastomeric materials. In this work, three naturally occurring amino acids, L-cystine, L-cysteine, and creatine, were investigated as alternative health-friendly sulfur vulcanization accelerators in NBR compounds filled with carbon black. Their influence on curing characteristics, crosslink density, morphology, mechanical performance, viscoelastic behavior, thermal stability, and thermo-oxidative aging resistance of NBR composites was systematically evaluated and compared with that of conventional accelerator systems. The amino acids effectively promoted sulfur crosslinking, although their activity depended strongly on molecular structure. Cystine and creatine exhibited the most promising performance, providing cure characteristics and mechanical properties comparable to those achieved with commercial accelerators while maintaining satisfactory thermal stability and aging resistance of carbon black-filled rubber composites. These findings demonstrate that naturally occurring amino acids constitute promising bio-derived and non-allergenic alternatives to conventional sulfur vulcanization accelerators and may contribute to the development of safer elastomeric materials with reduced reliance on potentially hazardous or allergenic substances.

## 1. Introduction

The increasing demand for environmentally responsible materials has accelerated the development of sustainable technologies for the production of advanced polymer and elastomer systems. Besides reducing the environmental footprint of polymer manufacturing, current research is increasingly focused on replacing hazardous chemicals with naturally derived, renewable, and biocompatible alternatives. Such an approach is consistent with the principles of green chemistry and the circular economy and has become particularly important for materials intended for prolonged contact with the human body. Consequently, the incorporation of naturally occurring molecules into polymer formulations has emerged as an effective strategy for improving both the safety and sustainability of advanced biomedical materials [1,2]. Among synthetic elastomers, acrylonitrile–butadiene rubber (NBR) occupies a prominent position in biomedical and healthcare applications [3,4] because of its excellent resistance to oils, chemicals, and biological fluids, together with high mechanical strength, abrasion resistance, and elasticity [5,6]. These properties have led to its widespread use in disposable and reusable medical gloves, pharmaceutical seals, tubing, gaskets, laboratory equipment, and numerous protective products. Unlike natural rubber latex, NBR does not contain allergenic proteins responsible for type I latex allergy, making it one of the preferred elastomeric materials for many medical applications. Nevertheless, despite its favorable performance, the overall safety of NBR products also depends on the chemical composition of the vulcanization system used during processing.

Sulfur vulcanization remains the dominant crosslinking technology for unsaturated elastomers because it produces three-dimensional polymer networks with excellent mechanical performance and long-term durability [7]. The efficiency of sulfur vulcanization is primarily controlled by organic accelerators, which significantly reduce curing time and temperature while determining the structure of the crosslinked network and, consequently, the final properties of the vulcanizates [8,9,10,11,12]. Among the most widely used commercial accelerators are tetramethylthiuram disulfide (TMTD), 2-mercaptobenzothiazole (MBT), and 1,3-diphenylguanidine (DPG), all of which have been successfully employed in industrial rubber technology for decades [12]. Despite their technological effectiveness, many conventional vulcanization accelerators have become a growing subject of toxicological and regulatory concern [12,13,14]. Thiurams and benzothiazoles are well-recognized causes of allergic contact dermatitis and are among the most frequently identified rubber additives responsible for delayed hypersensitivity reactions [13]. Moreover, several sulfur accelerator systems may generate nitrosamines or other potentially hazardous degradation products during processing or service, while the migration of low-molecular-weight additives from elastomeric products remains an additional concern, particularly for biomedical devices and personal protective equipment. Consequently, the search for safer and more health-friendly curing systems has become an important research direction in rubber science.

Naturally occurring amino acids represent an attractive class of bio-derived compounds that could contribute to the development of greener vulcanization technologies. Owing to their renewable origin, low toxicity, biodegradability, and excellent biocompatibility, amino acids have attracted increasing attention as functional components of biomedical materials, drug delivery systems, hydrogels, and tissue-engineering scaffolds. Their molecular structures contain various reactive functional groups capable of participating in acid–base interactions, metal coordination, and sulfur chemistry. In particular, sulfur-containing amino acids, such as L-cystine and L-cysteine, possess disulfide or thiol groups that may influence sulfur crosslinking reactions, whereas amino-containing molecules such as creatine may modify the kinetics of accelerator activation and network formation [12].

Previous studies concerning sustainable rubber technology have primarily focused on the incorporation of renewable fillers, bio-based plasticizers, vegetable oils, lignin, cellulose, or other naturally derived additives into elastomeric matrices [15,16,17]. In contrast, considerably less attention has been devoted to replacing conventional sulfur vulcanization accelerators which cause allergic effects with naturally occurring biomolecules while maintaining satisfactory curing efficiency and material performance. The development of such bio-based curing systems could simultaneously improve the toxicological profile of rubber products and reduce dependence on conventional petrochemical additives without compromising their functional properties. In our previous work [12], we conducted a comprehensive review of the available literature, which revealed that amino acids had been investigated predominantly as anti-aging agents or as components of binary curing systems for rubber vulcanization [18,19,20,21]. To address this research gap, we have reported, for the first time, the use of biologically active amino acids as alternative sulfur vulcanization accelerators for acrylonitrile–butadiene rubber (NBR). This study has demonstrated that amino acids, although they did not shorten the vulcanization time compared with conventional accelerators, positively influenced the crosslinking process by stabilizing sulfur crosslinks, increasing the enthalpy of vulcanization, and maintaining an appropriate crosslink density of the vulcanizates. Moreover, selected amino acids, particularly L-cystine, L-cysteine and creatine, beneficially affected the mechanical performance, thermo-oxidative aging resistance, thermal stability, and dynamic properties of the resulting vulcanizates. These findings indicated that naturally occurring amino acids can serve as promising, environmentally benign curing additives and established the basis for further investigation of their practical applicability in technologically relevant rubber formulations.

Based on these encouraging results, we focused on the amino acids that exhibited the highest curing activity and the most favorable influence on the performance of unfilled NBR vulcanizates. Since commercial rubber compounds almost invariably contain reinforcing fillers that strongly affect curing behavior, filler–rubber interactions, crosslink structure, and the final functional properties, it is essential to verify whether the beneficial effects observed in unfilled rubber systems are retained in reinforced formulations. Carbon black, as the most widely used reinforcing filler in industrial rubber technology, may substantially modify the activity of bio-based accelerators and the resulting network architecture [22,23]. Because carbon black strongly influences vulcanization kinetics, filler–rubber interactions, crosslink distribution, and the architecture of the elastomeric network, the performance of amino acid-based curing systems must be verified in reinforced compounds before their practical applicability can be assessed.

Therefore, the objective of the present study was to investigate the applicability of three naturally occurring amino acids, L-cystine, L-cysteine, and creatine, as alternative vulcanization accelerators for carbon black-filled NBR composites. Their influence on curing characteristics, crosslink density, morphology, mechanical properties, viscoelastic behavior, thermal stability, and thermo-oxidative aging resistance was systematically evaluated and compared with that of conventional accelerators. The results provide new insights into the use of naturally derived molecules as functional curing additives in filled elastomer systems and demonstrate their potential for developing more sustainable elastomeric materials, with reduced use of conventional vulcanization additives that may be associated with allergenic or adverse health effects.

## 2. Results and Discussion

### 2.1. Vulcanization Characteristics and Crosslink Density of NBR Composites

**Figure 1:**
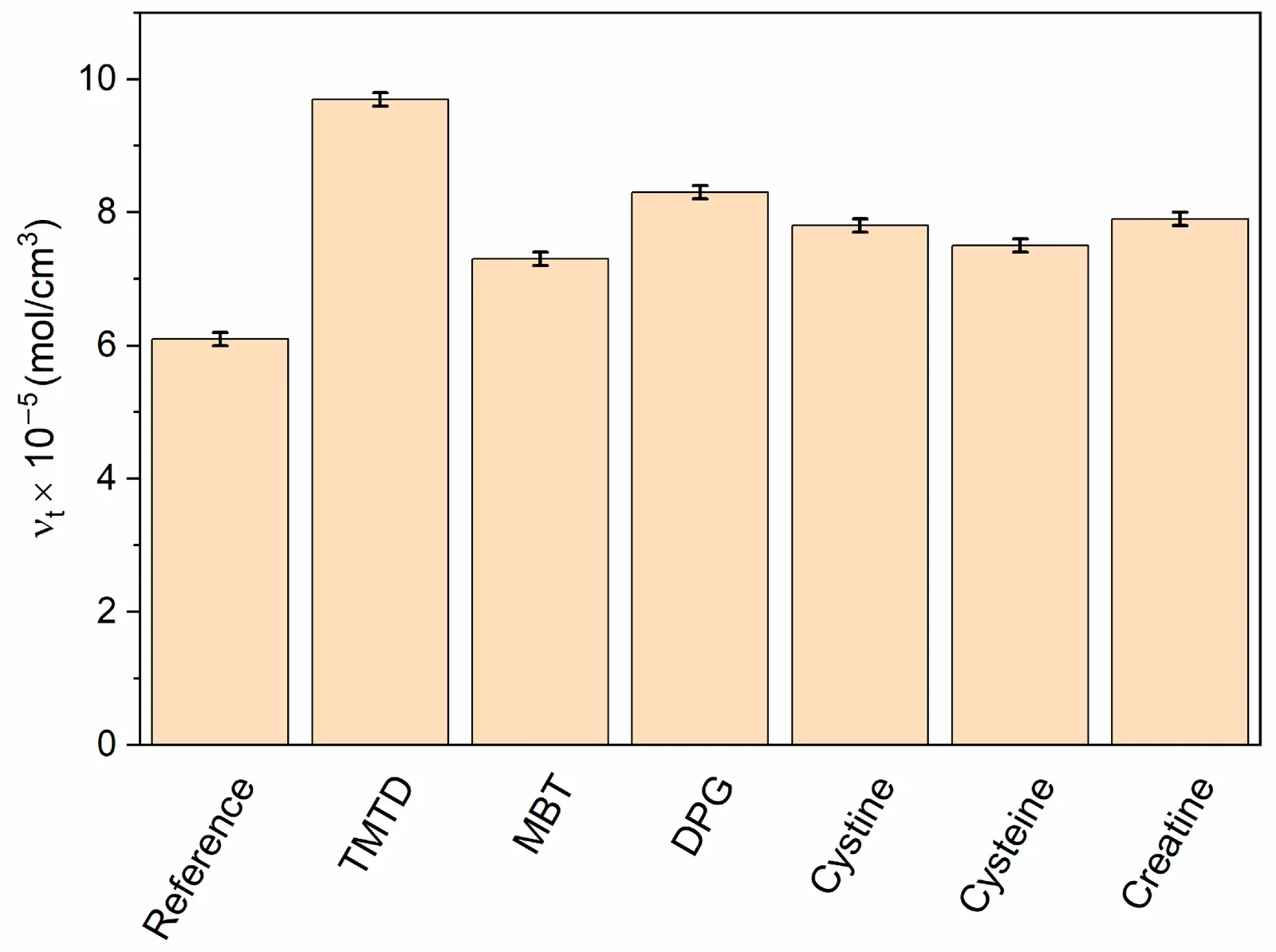
Crosslink density of carbon black-filled NBR vulcanizates.

Carbon black is not a chemically inert component of the rubber compound. Its high specific surface area and the presence of oxygen-containing surface groups enable the adsorption of curing system components, such as accelerators, activators, and other low-molecular-weight additives. Consequently, carbon black may influence the vulcanization kinetics as well as the rheometric properties of the compound [11]. On the other hand, amino acids contain amino (-NH_2_) and carboxyl (-COOH) functional groups, while some, such as L-cysteine and L-cystine, also possess thiol (-SH) or disulfide (-S-S-) groups. These compounds may adsorb onto the surface of carbon black through hydrogen bonding or acid–base interactions, interact with ZnO and stearic acid, participate in the formation of zinc complexes, affect the dispersion of curing system components, and modify the formation of sulfur crosslinks [12]. Therefore, the activity of amino acids observed in the unfilled NBR may not be identical to that in carbon black-filled composites. For this reason, we considered it justified to evaluate the rheometric parameters of NBR composites filled with carbon black, as well as to investigate their curing behavior. The rheometric parameters are summarized in Table 1, while the values of crosslink density of vulcanizates are presented in Figure 1.

The rheometric data (Table 1) and crosslink density measurements (Figure 1) indicated that the investigated amino acids promoted the sulfur vulcanization of carbon black-filled NBR compounds as compared to the reference rubber compound without accelerator. However, their efficiency was strongly dependent on their molecular structure. The minimum torque during rheometric measurement (S_min_), reflecting the viscosity of the uncured rubber compounds, remained within a narrow range of 1.1–1.3 dNm irrespective of the accelerator used. This indicates that replacing conventional accelerators with amino acids did not significantly influence the processability of the carbon black-filled rubber compounds. The relatively small differences in S_min_ further suggest that the viscosity before vulcanization is governed primarily by the presence of filler rather than by the chemical nature of the accelerator. Compared with the unfilled NBR compounds investigated in our previous work [12], in which S_min_ was 0.6–0.7 dNm, the carbon black-filled formulations exhibited higher initial torque values due to the reinforcing effect of the filler, i.e., carbon black, which increased the viscosity of uncured rubber compounds by restricting polymer chain mobility and forming a filler network [24]. Therefore, the effect of filler on the viscosity of the rubber compounds as compared to the unfilled NBR was visible.

The reference rubber compound containing sulfur without any accelerator exhibited the characteristic behavior of an unaccelerated sulfur curing system. The relatively long scorch time (t_5_ = 9 min) reflected a stable curing system with a low tendency toward premature crosslinking during the initial heating stage. On the other hand, prolonged optimal vulcanization time (t_90_ = 95 min) indicated a very slow rate and kinetics of sulfur vulcanization. Furthermore, the reference vulcanizate exhibited the lowest crosslink density, which was 6.1 × 10^−5^ mol × cm^−3^ (Figure 1), confirming the limited efficiency of network formation under these conditions. This corresponded to a slightly lower torque increase (∆S) compared to the other carbon black-filled NBR compounds. Consistent with our previous findings [12] and the established mechanism of sulfur vulcanization [25], the curing process in the reference rubber compound probably occurred mainly via direct sulfur crosslinking of the unsaturated rubber chains rather than through an accelerator-derived active sulfurating complex. As expected, the incorporation of commercial accelerators markedly improved the curing characteristics. The NBR compound cured using TMTD exhibited the highest curing activity, reducing significantly the scorch time (t_5_ = 1 min) and optimal vulcanization time to 9 min. This behavior resulted from the dual function of TMTD, which acts both as an ultra-accelerator and as a sulfur donor, increasing the availability of reactive sulfur species during vulcanization and promoting rapid formation of a dense crosslinked network. Simultaneously, the NBR compound with TMTD achieved the highest value of ∆S = 15.2 dNm, and consequently the highest crosslink density (9.7 × 10^−5^ mol × cm^−3^) of the vulcanizate, confirming the formation of the most densely crosslinked elastomer network. NBR compounds containing MBT and DPG, which belong to the groups of medium- and slow-acting accelerators, respectively, exhibited lower ∆S (9.3 dNm and 12.6 dNm, respectively), which was also associated with the lower crosslink densities of the vulcanizates as compared to vulcanizate with TMTD. The molecular structures of those accelerators (Figure 9) show that they are not sulfur donors; consequently the formation of reactive sulfurating intermediates is less extensive than in the TMTD-containing curing system, leading to a lower crosslinking efficiency. Moreover, as expected, the optimal vulcanization times of the carbon black-filled NBR compounds followed the accelerating activity of the conventional curing systems, increasing in the order TMTD < MBT < DPG (t_90_ of 9 min, 30 min and 39 min, respectively). However, it is also worth mentioning that MBT efficiently initiated vulcanization but produced a less developed elastomer network due to the low value of crosslink density, which was 7.3 × 10^−5^ mol × cm^−3^ (Figure 1). The relatively low ∆S obtained for MBT may be associated with interactions between this accelerator and the carbon black surface. An aromatic benzothiazole structure and polar functional groups of MBT accelerator may induce a partial adsorption of benzothiazole species on the carbon black surface. Such interactions may decrease the availability of the accelerator for the formation of active sulfurating intermediates and consequently may reduce the efficiency of the curing system [26,27]. Such interactions have been reported for carbon black-filled rubber compounds and may partially explain the lower crosslink density observed for MBT-containing vulcanizate compared with DPG despite its higher intrinsic curing activity.

The amino acid-containing NBR compounds filled with carbon black exhibited curing characteristics intermediate between those of the reference rubber compound and conventionally accelerated systems. In contrast to commercial accelerators, the amino acids, such as cystine, cysteine and creatine, provided considerably longer scorch and optimal vulcanization times. Among them, creatine was the most active during the crosslinking process, reducing t_90_ to 70 min and producing the highest ∆S of 13.1 dNm, together with the highest crosslink density (7.9 × 10^−5^ mol × cm^−3^) among all NBR compounds containing amino acids. Moreover, creatine resembles the chemical structure of DPG and may facilitate the formation of active sulfurating species through interactions with Zn^2+^ ions originating from ZnO. Rubber composites containing cystine and cysteine exhibited similar curing behavior, with t_90_ of 81 min and 78 min, respectively, and intermediate crosslink densities (7.5 and 7.8 × 10^−5^ mol × cm^−3^, respectively). Cystine, containing a disulfide bridge, produced a slightly higher ∆S and crosslink density than cysteine. Cysteine, owing to the presence of the thiol group, may also participate in sulfur transfer reactions; however, its accelerating activity remained lower than that of the commercial thiazole accelerator MBT. These results indicated that although amino acids are less efficient than conventional accelerators in promoting sulfur vulcanization, they are capable of producing well-developed elastomer crosslinks in carbon black-filled NBR compounds.

The investigated amino acids were selected based on their molecular structures and their structural similarity to the commercial vulcanization accelerators evaluated in this study. As demonstrated in our previous work [12], the influence of amino acids on the curing behavior and vulcanization kinetics is closely related to their molecular structure and the presence of functional groups analogous to those occurring in conventional accelerators. The present study extends this concept to carbon black-filled NBR compounds, in which filler–curative interactions may additionally affect the crosslinking process and the activity of accelerators. Compared with our previous study on unfilled NBR compounds, an important difference can be observed. In the absence of carbon black, the curing characteristics reflected primarily the intrinsic chemical activity of the investigated amino acids. In the present carbon black-filled systems, however, the curing behavior is additionally influenced by competitive interactions occurring between the curing agents, ZnO, sulfur and the active surface of carbon black. Adsorption of low-molecular-weight curing agents on carbon black may reduce their availability for the vulcanization reaction, particularly in the case of polar accelerators such as MBT [28]. Nevertheless, despite these additional interactions, the overall trends remained consistent with those reported previously. Creatine remained the most active amino acid, whereas cystine and cysteine exhibited intermediate curing activity, demonstrating that amino acids retain their accelerating effect even in the presence of an active reinforcing filler.

Overall, the rheometric and crosslink density results indicate that amino acids cannot compete with conventional accelerators in terms of curing rate. However, they significantly improve the efficiency of sulfur vulcanization compared with the unaccelerated system while providing substantially longer scorch times, thereby improving processing safety. Importantly, the use of amino acids instead of conventional vulcanization accelerators may reduce the presence of low-molecular-weight chemicals known to induce allergic contact dermatitis. Consequently, amino acid-based curing systems may be of interest for rubber products in which the use of allergenic and potentially harmful substances is intended to be minimized.

**Figure 2:**
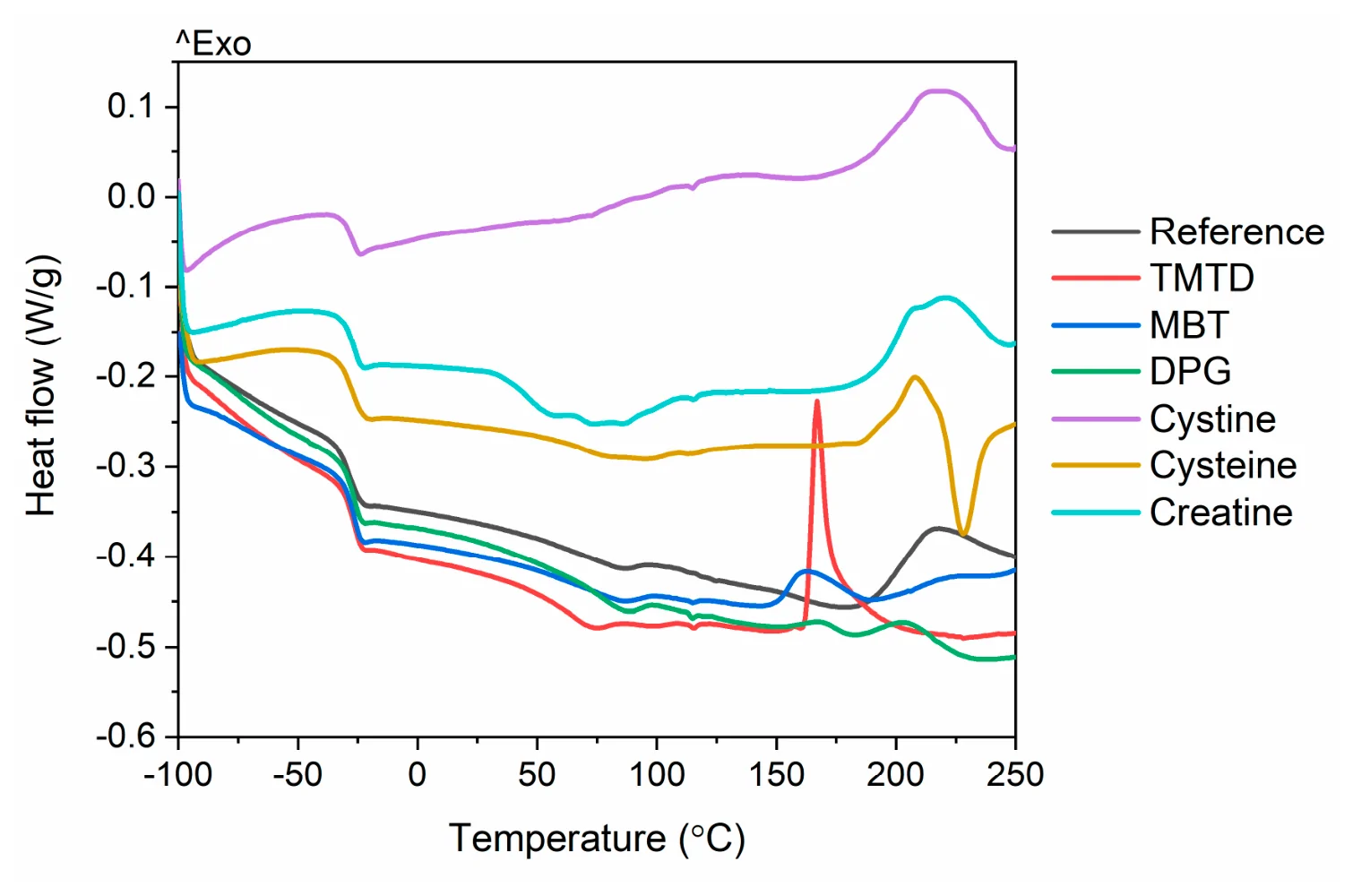
Curing curves of carbon black-filled NBR compounds determined by differential scanning calorimetry (DSC).

As mentioned, carbon black has been reported to influence the kinetics, conversion, and thermal behavior of sulfur vulcanization owing to its surface activity and interactions with curing agents [29,30]. Therefore, differential scanning calorimetry (DSC) was employed as a complementary technique to rheometric measurements to provide additional information on the thermal characteristics of the crosslinking reactions. While rheometry describes the curing kinetics under isothermal conditions, DSC provides insight into the temperature range and thermal effect of vulcanization [31]. The combined results of both techniques enable a comprehensive evaluation of the influence of the investigated amino acids on the sulfur crosslinking of elastomers. The DSC results are presented in Table 2, whereas the DSC curves are shown in Figure 2.

The glass transition temperature (T_g_) remained nearly unchanged for all investigated compounds (−27 to −28 °C), indicating that neither the commercial accelerators nor the amino acids affected the segmental mobility of the NBR chains before the onset of vulcanization (Table 2). This observation confirmed that the investigated additives primarily influenced the curing reaction rather than the physical state of the uncured rubber. Significant differences were observed in the temperature range of the crosslinking reaction. The reference rubber compound without any accelerator exhibited the highest range of the crosslinking temperature (192–247 °C), confirming the relatively low reactivity of the unaccelerated sulfur curing system. This result is fully consistent with the rheometric measurements, which showed the longest t_90_ of 95 min for this rubber compound. The incorporation of conventional accelerators considerably shifted the crosslinking process towards lower temperatures. TMTD, classified as an ultra-fast accelerator, exhibited a sharp exothermic peak of crosslinking within the narrow temperature range of 163–173 °C and exhibited a relatively high crosslinking enthalpy (14.6 J g^−1^), which confirmed rapid and efficient network formation due to the highest crosslink density of that vulcanizate. Regarding TMTD, the sharp and relatively narrow exothermic peak is consistent with the very rapid vulcanization promoted by this accelerator. TMTD belongs to the class of ultra-accelerators, meaning it possesses exceptionally high reactivity. Once the activation temperature is reached, the crosslinking reaction occurs almost instantaneously. It is also consistent with its rapid vulcanization kinetics. A similar DSC behavior was observed for TMTD in our previous study [12], where crosslinking occurred within a narrow temperature range and was accompanied by a relatively high enthalpy. On the other hand, MBT initiated crosslinking at the lowest temperature (156 °C), although the reaction proceeded over a broader temperature interval (156–185 °C) and was accompanied by the lowest enthalpy of reaction (4.6 J g^−1^). This suggested that MBT efficiently initiates sulfur activation, but the overall thermal effect of network formation was considerably lower than that observed for TMTD, which also confirmed the lowest crosslink density of the vulcanizate with MBT. In contrast, DPG, representing a slow accelerator, initiated crosslinking at a higher temperature (177 °C), and the whole curing reactions proceeded over the temperature range of 177–221 °C with an enthalpy comparable to that of MBT (4.3 J g^−1^), which reflected lower curing activity of DPG. This wide exothermic peak observed for DPG between approximately 177 and 221 °C may reflect the more gradual and complex course of the crosslinking process characteristic of this slow-acting accelerator. A similar thermal behavior of the DPG-containing system was also observed in our previous study [12]. In the case of NBR compounds with amino acid-based curing systems, the network formation occurred over broader temperature intervals and generally at higher temperatures than in the conventionally accelerated vulcanization. Among the investigated amino acids, cystine was the most active, exhibiting both the lowest onset temperature of crosslinking (172 °C) and the highest enthalpy of vulcanization (16.8 J g^−1^). This behavior may be attributed to the presence of the disulfide bond, which may participate in sulfur exchange reactions during crosslinking. Cysteine initiated crosslinking at a slightly higher temperature (179 °C), whereas creatine showed intermediate thermal behavior (175 °C) and a relatively high enthalpy of vulcanization (14.8 J g^−1^). In addition to the exothermic peak associated with vulcanization, the DSC curve of the NBR compound containing cysteine (Figure 2) exhibited an additional endothermic transition in the temperature range of 216–240 °C, which was due to the melting of cysteine. Overall, the DSC results demonstrate that although amino acids did not reduce the crosslinking temperature as effectively as commercial accelerators, they promoted efficient network formation over a wider temperature range.

Moreover, the DSC results were in good agreement with the rheometric measurements. While the prolonged values of t_90_ obtained for the amino acid-containing compounds clearly indicated slower cure kinetics under isothermal conditions, the relatively high enthalpy of crosslinking demonstrated that these systems are still capable of forming a well-developed elastomer network.

The curing behavior of the amino acid-modified NBR compounds was closely related to both the molecular structure and functional groups of the investigated biomolecules (Figure 2) and their interactions within the carbon black-filled curing system. Besides participating in sulfur vulcanization, amino acids may interact with the carbon black surface through hydrogen bonding and acid–base interactions involving their amino and carboxyl groups. Carbon black may also adsorb low-molecular-weight curatives, thereby affecting their availability during vulcanization. Consequently, the curing behavior depended not only on the intrinsic activity of the amino acids but also on their interactions with carbon black, ZnO, stearic acid, and sulfur [29,30]. Nevertheless, all investigated amino acids remained active in the carbon black-filled NBR compounds, effectively promoting sulfur crosslinking despite the presence of the reinforcing filler.

### 2.2. Scanning Electron Microscopy (SEM) of NBR Vulcanizates

**Figure 3:**
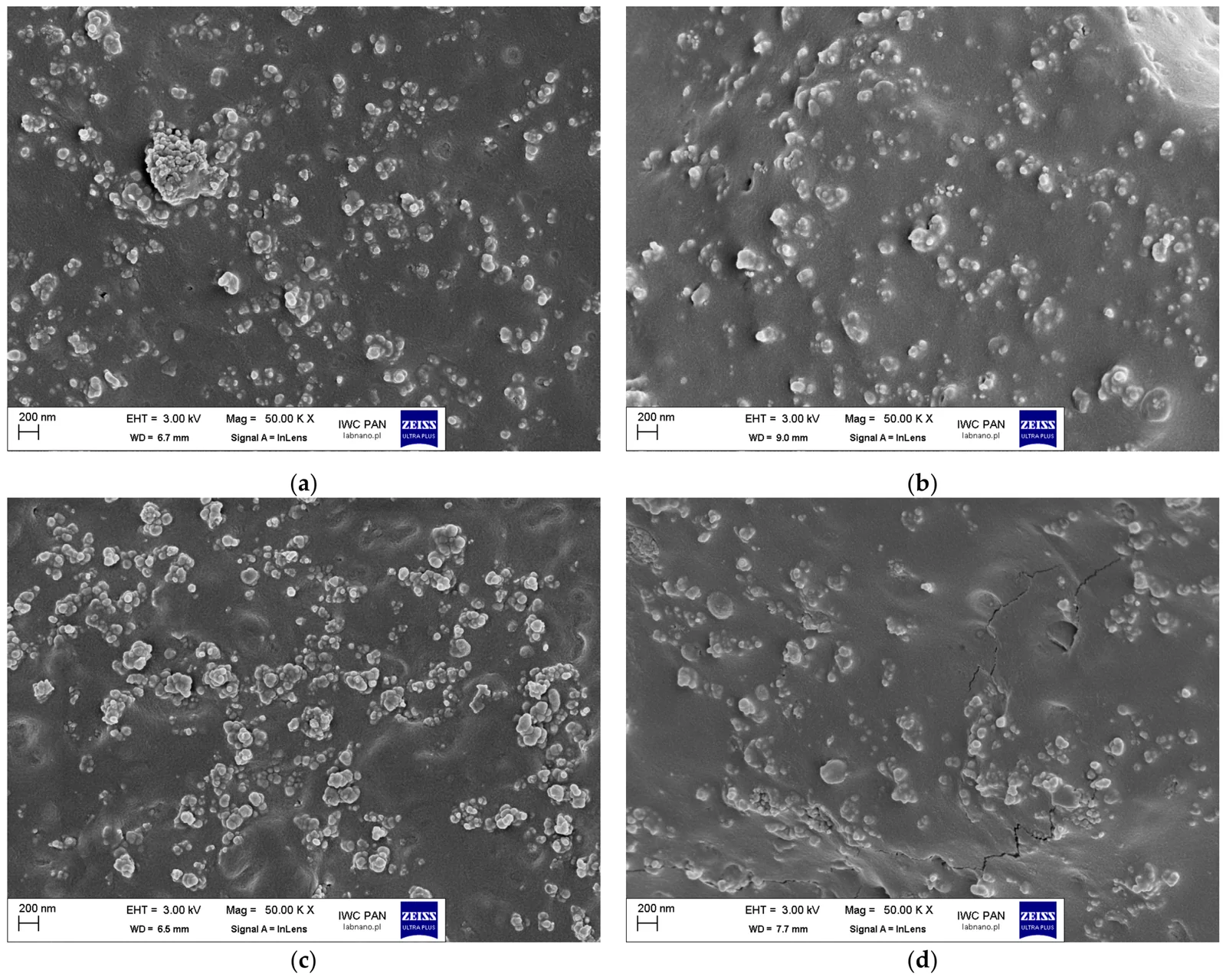

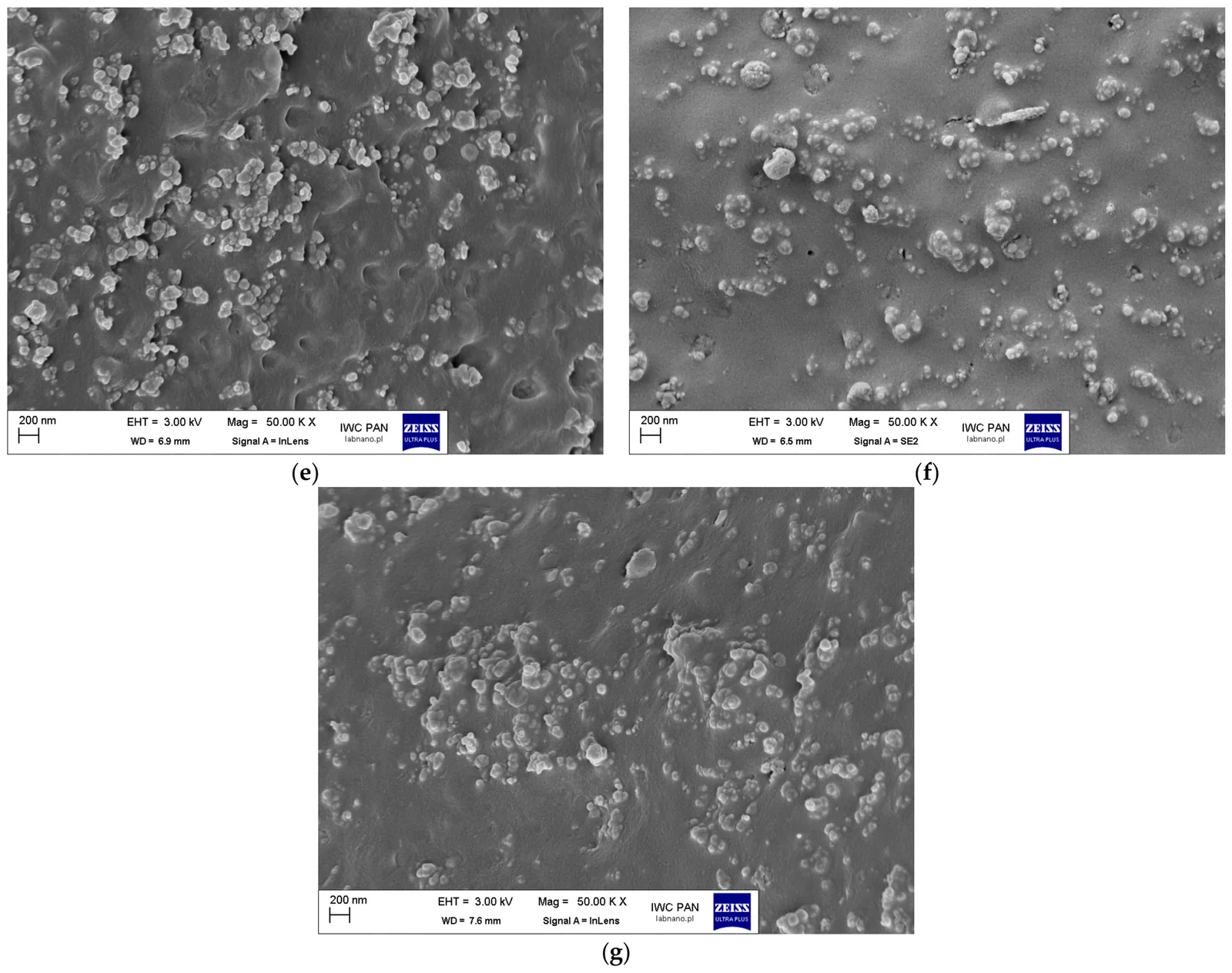
Scanning electron microscopy (SEM) images of carbon black-filled NBR vulcanizates: (**a**) reference vulcanizate without accelerators; (**b**) vulcanizate with TMTD; (**c**) vulcanizate with MBT; (**d**) vulcanizate with DPG; (**e**) vulcanizate with cystine; (**f**) vulcanizate with cysteine; (**g**) vulcanizate with creatine.

Carbon black filler is commonly introduced into rubber compounds at high loadings to ensure effective reinforcement. Owing to its strong tendency to form aggregates and agglomerates, uniform dispersion of the filler during compounding is not always achieved, which may significantly influence the curing process and the final properties of the vulcanizates. Therefore, scanning electron microscopy (SEM) was employed to assess the dispersion of carbon black particles and the curing system components within the NBR matrix. This analysis allowed the influence of filler morphology to be distinguished from the effects associated with the curing system itself. The corresponding SEM micrographs are shown in Figure 3.

The SEM micrographs (Figure 3) revealed that carbon black particles were well dispersed throughout the NBR matrix irrespective of the curing system employed. In all vulcanizates, the filler was present predominantly as finely dispersed aggregates that were homogeneously distributed within the elastomer matrix, while no large agglomerates or filler-rich domains were observed. This indicates that the applied two-stage mixing procedure ensured efficient dispersion and satisfactory incorporation of the reinforcing filler. Furthermore, the absence of noticeable phase separation or filler agglomeration suggests that the observed differences in curing behavior and vulcanizate properties originated primarily from the curing systems rather than from variations in filler dispersion.

The reference vulcanizate (Figure 3a) exhibited a relatively homogeneous morphology with uniformly distributed carbon black aggregates and only occasional larger agglomerates. The NBR vulcanizate with TMTD showed a similar microstructure but with a slightly more uniform aggregate distribution, suggesting efficient filler dispersion and good wetting of the carbon black particles by the rubber matrix.

The MBT- and DPG-containing vulcanizates (Figure 3c,d) also displayed homogeneous morphologies without pronounced filler agglomeration. Although isolated clusters of carbon black were occasionally visible, especially in the DPG-containing vulcanizate (Figure 3d), no pronounced carbon black agglomeration or phase separation was detected in this sample. Therefore, these features are unlikely to be associated with poor filler dispersion and may instead be related to local variations in the formed crosslinks and stress concentration during cryogenic fracture. It should also be noted that the SEM specimens were cryogenically fractured prior to analysis, and hence the observed cracks may be partly related to the fracture procedure itself. Moreover, their size and frequency remained low, indicating that the commercial accelerators did not adversely affect filler dispersion during compounding.

The amino acid-containing vulcanizates (Figure 3e–g) exhibited microstructures comparable to those of the reference and conventionally accelerated composites. No substantial deterioration in carbon black dispersion was observed after replacing the commercial accelerators with cystine, cysteine, or creatine. Among these systems, the creatine-containing composite (Figure 3g) appeared to possess the most uniform distribution of carbon black aggregates, whereas vulcanizate with cystine (Figure 3e) contained several locally denser filler clusters. Nevertheless, these agglomerates remained relatively small, and no extensive filler networking or phase separation was detected.

Overall, the SEM observations indicated that the incorporation of amino acids into the curing system did not negatively influence the morphology of the carbon black-filled NBR composites. Since carbon black particles have a strong tendency to form aggregates and agglomerates, achieving a homogeneous filler distribution in elastomer compounds is generally difficult [32,33]. Therefore, the relatively homogeneous dispersion observed in the present study indicates the effective incorporation of the reinforcing filler and curing system components into the NBR matrix. A high degree of carbon black dispersion is expected to contribute to the enhanced mechanical properties of the vulcanizates.

### 2.3. Mechanical Properties of NBR Vulcanizates Under Static and Dynamic Conditions

The SEM analysis demonstrated that the incorporation of amino acids into the curing system did not adversely affect the morphology of the carbon black-filled NBR composites. In all investigated formulations, carbon black particles exhibited a relatively homogeneous dispersion throughout the rubber matrix. Since the differences in filler dispersion were negligible, it was necessary to evaluate the mechanical performance of the obtained vulcanizates to determine how the different accelerating systems influenced their strength and deformation behavior under static conditions. The results of the tensile properties and hardness measurements are presented in Table 3.

As shown in Table 3, the type of accelerator in the curing system significantly influenced the mechanical performance of the NBR vulcanizates filled with carbon black. The stress at 100% elongation (SE_100_), commonly regarded as an indicator of elastomer network stiffness, depended on the curing system employed. The reference vulcanizate exhibited an intermediate SE_100_ value of 2.0 MPa, corresponding to its relatively low crosslink density. Among the conventionally accelerated systems, TMTD produced the vulcanizate with the highest SE_100_ (3.3 MPa), consistent with its highest torque increment and crosslink density, whereas the vulcanizate with DPG also exhibited relatively high stiffness of 2.6 MPa. In contrast, the MBT-containing vulcanizate showed SE_100_ of 2.0 MPa, comparable to that of the reference vulcanizate despite its higher crosslink density, suggesting that the initial stiffness depends not only on the crosslink density but also on the architecture of the sulfur network. Among NBR composites containing amino acid-based curing systems, creatine provided the highest SE_100_, followed by cysteine and cystine (2.4 MPa, 2.1 MPa and 2.0 MPa, respectively). This trend was consistent with the corresponding crosslink densities of the vulcanizates, confirming that creatine produced the stiffest network among the investigated amino acids. Nevertheless, all amino acid-containing vulcanizates exhibited lower SE_100_ values than the TMTD-cured compound, but the stiffness was comparable to those cured with MBT and DPG.

The tensile strength (TS) also correlated with the crosslink density of NBR vulcanizates. The highest TS of approximately 18 MPa was obtained for vulcanizates with DPG and TMTD, indicating that the ultimate strength depends not only on the crosslink density but also on the architecture of the elastomer network and dispersion of the filler. The amino acid-containing vulcanizates exhibited TS values between 13.9 and 15.3 MPa, comparable to those of the reference vulcanizate and that containing MBT. The highest TS among the investigated amino acids was provided by the curing system with creatine. The results indicated that curing systems based on amino acids are capable of providing vulcanizates with mechanical properties comparable to those obtained using conventional vulcanization accelerators.

Considering the results of elongation at break (EB), it was observed that NBR vulcanizate with TMTD exhibited the lowest EB of 348%, and consequently the lowest elasticity, resulting from the most densely crosslinked elastomer network. This value was also significantly lower than for the reference vulcanizate without any accelerator (EB of 466%). This interpretation is further supported by its highest hardness, indicating reduced chain mobility and lower deformability under applied stress. NBR vulcanizate with MBT exhibited a considerably higher EB than vulcanizate with TMTD, approximately 560%. It was accompanied by slightly lower hardness compared to that of the TMTD-containing vulcanizate. These results suggest that the network formed in the presence of MBT allowed greater molecular mobility during deformation, enabling the material to sustain higher strains before failure. A similar tendency was observed for the vulcanizate with DPG.

Regarding the carbon black-filled NBR vulcanizates with amino acids, they exhibited EB comparable to those provided by the conventional accelerating systems based on DPG and MBT. The high EB was also combined with relatively high hardness (63–64 Shore A), confirming the good flexibility of the crosslinked elastomer network.

Most importantly, replacing conventional vulcanization accelerators with amino acids did not result in significant deterioration of the mechanical performance of the NBR vulcanizates filled with carbon black.

**Figure 4:**
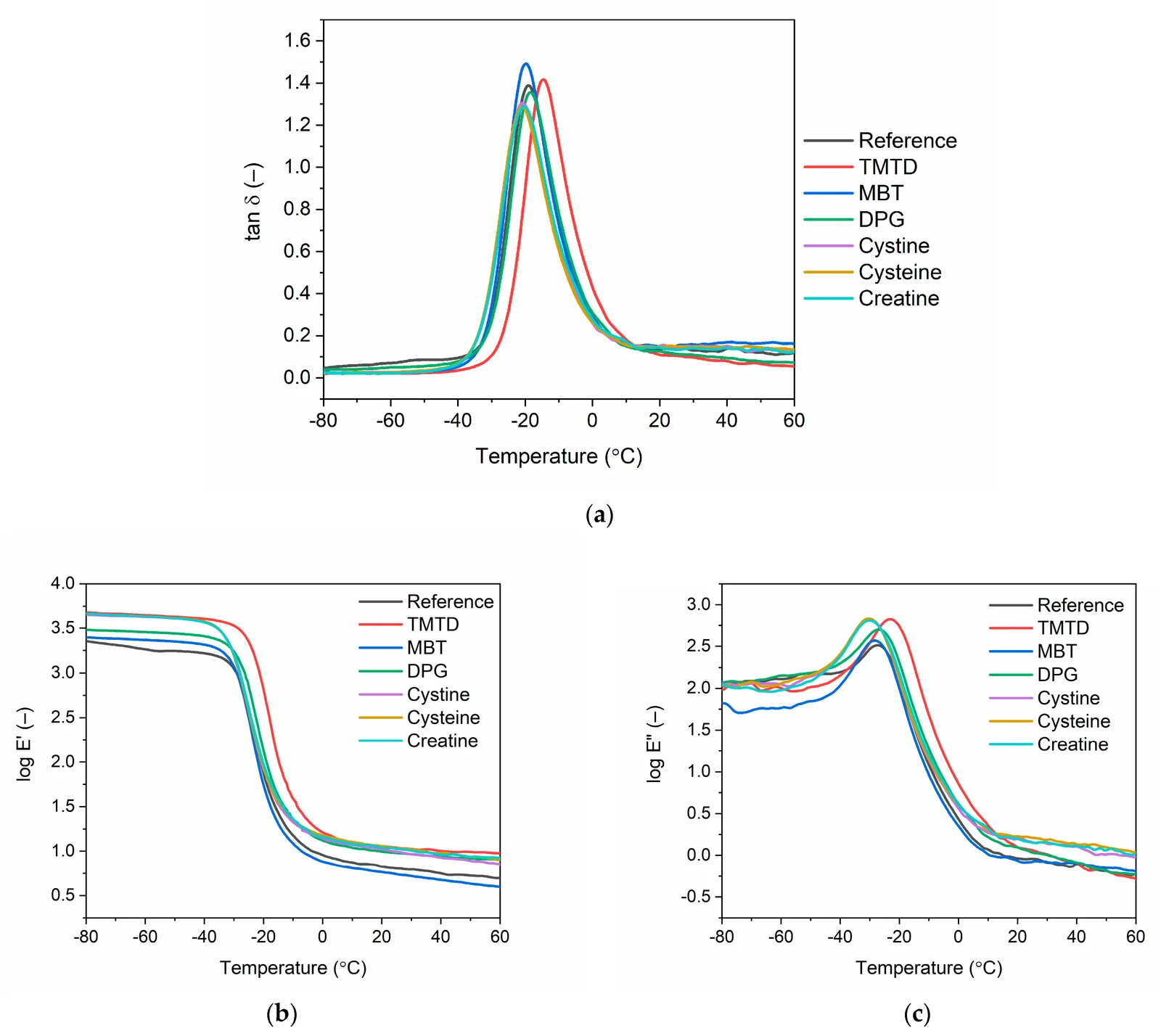
DMA curves as a function of temperature for NBR vulcanizates: (**a**) loss factor (tan δ); (**b**) storage modulus (E’); (**c**) loss modulus (E”).

To evaluate the effect of the investigated curing systems on the viscoelastic behavior of NBR vulcanizates, dynamic mechanical analysis (DMA) was performed as a function of temperature. Since the dynamic mechanical response of filled elastomers depends on both the crosslinked network and filler–rubber interactions, DMA provides complementary information on the influence of amino acids on the structure and performance of carbon black-filled NBR composites as a function of temperature. The storage modulus (E′) and loss modulus (E″) further describe the elastic and dissipative components of the viscoelastic response, respectively. Differences between the formulations were evaluated under the applied frequency and temperature conditions. The obtained results are summarized in Table 4, while the temperature dependence of the mechanical loss factor (tan δ), storage modulus and loss modulus (E′ and E″, respectively) is presented in Figure 4.

The tan δ curves revealed a single relaxation peak for all vulcanizates (Figure 4a), corresponding to the glass transition of the NBR matrix. The α-relaxation peak observed in the tan δ curves is associated with the segmental mobility of the NBR chains and reflects the transition of the elastomer from the glassy to the high-elastic state [34]. Therefore, T_g_ determined from the tan δ maxima ranged from −21 to −15 °C (Table 4), indicating only minor differences in the segmental mobility of the polymer chains among the investigated vulcanizates under the applied DMA conditions. Nevertheless, NBR vulcanizates with commercial accelerators showed slightly higher T_g_ in dynamic conditions. T_g_ decreased in the order of TMTD (−15 °C), DPG (−18 °C), and MBT (−20 °C), suggesting enhanced flexibility of elastomer chains at low temperatures. The obtained results were also in good agreement with the increasing crosslink density, exhibiting the same trend as the vulcanizates containing commercial accelerators. NBR vulcanizate with TMTD exhibited a noticeable shift in the maximum tan δ peak at T_g_ towards higher temperatures (−15 °C), which was consistent with its highest crosslink density determined by equilibrium swelling. The higher crosslink density may restrict the mobility of polymer chains, contributing to the shift in T_g_ toward higher temperatures. In contrast, NBR compounds cured in the presence of amino acids demonstrated T_g_ values between −20 and −21 °C, closely resembling those of the reference rubber compound, which was also correlated to their lower crosslink density and therefore probably better mobility of elastomer chains. The use of amino acids resulted in only minor differences in the tan δ values at T_g_ compared with the conventional accelerator systems. The vulcanizates containing conventional accelerators exhibited tan δ values in the range of 1.4–1.5, whereas all vulcanizates containing amino acids showed a tan δ value of 1.3. Additionally, compared with the unfilled NBR vulcanizates reported in our previous study [12], the carbon black-filled vulcanizates exhibited lower tan δ at T_g_. This behavior was attributed to the reinforcing effect of carbon black filler, which restricts the mobility of polymer chains through strong polymer–filler interactions and the formation of bound rubber. According to Robertson et al. [23], as a result, the elastic response of the material increases, while the energy dissipated during the glass transition decreases, leading to a lower tan δ peak. Under the applied DMA measurement conditions, the differences in the tan δ among the investigated NBR vulcanizates were relatively minor, considering the experimental uncertainty. However, these differences became more pronounced at higher temperatures corresponding to their potential operating conditions. NBR vulcanizates with amino acids exhibited higher tan δ values, especially at 60 °C (Table 4), indicating a greater contribution of the dissipative component to the viscoelastic response under the applied testing conditions. It may be beneficial for applications in which effective vibration dissipation under elevated-temperature conditions is required. The higher tan δ values may be associated with reversible hydrogen bonding interactions involving amino acid functional groups, which may contribute to mechanical energy dissipation during dynamic deformation [35]. However, while the effect of amino acids on the vibration damping performance of NBR compounds was clearly observed for the unfilled vulcanizates [12], the differences in tan δ values became much less pronounced in the carbon black-filled systems. This behavior is likely associated with the formation of bound rubber and the filler network, which restrict the mobility of polymer chains and increase the elastic response of the material. Consequently, these reinforcing effects may partially mask the influence of amino acids, as well as the effect of differences arising from variations in crosslink density.

Additionally, the storage modulus (E′) and loss modulus (E″) (Figure 4b,c) provide complementary information on the elastic and dissipative components of the viscoelastic response, respectively. As shown in Figure 4b, E′ decreased markedly near T_g_ for all vulcanizates, reflecting the glass-to-rubber transition and increased segmental mobility with increasing temperature. The highest E′ values were observed for the TMTD-cured vulcanizate, followed by the DPG-containing system, consistent with their higher crosslink densities and more constrained network structures. The amino acid-based vulcanizates showed intermediate E′ values, indicating less restrictive but sufficiently developed networks. Above T_g_, the differences in E′ decreased, suggesting that both crosslinking and carbon black reinforcement contributed to the dynamic stiffness of the filled NBR vulcanizates [35].

The E″ profiles (Figure 4c) exhibited distinct maxima near T_g_, corresponding well with the T_g_ values determined from the peak in the tan δ curves and reflecting energy dissipation associated with segmental relaxation of the NBR chains. The relatively similar E″ profiles indicate that the curing system had a limited effect on the dissipative component, while the response in the glass transition region was mainly governed by the α-relaxation of the NBR matrix [35].

### 2.4. Resistance to Thermo-Oxidative Aging of NBR Vulcanizates

**Figure 5:**
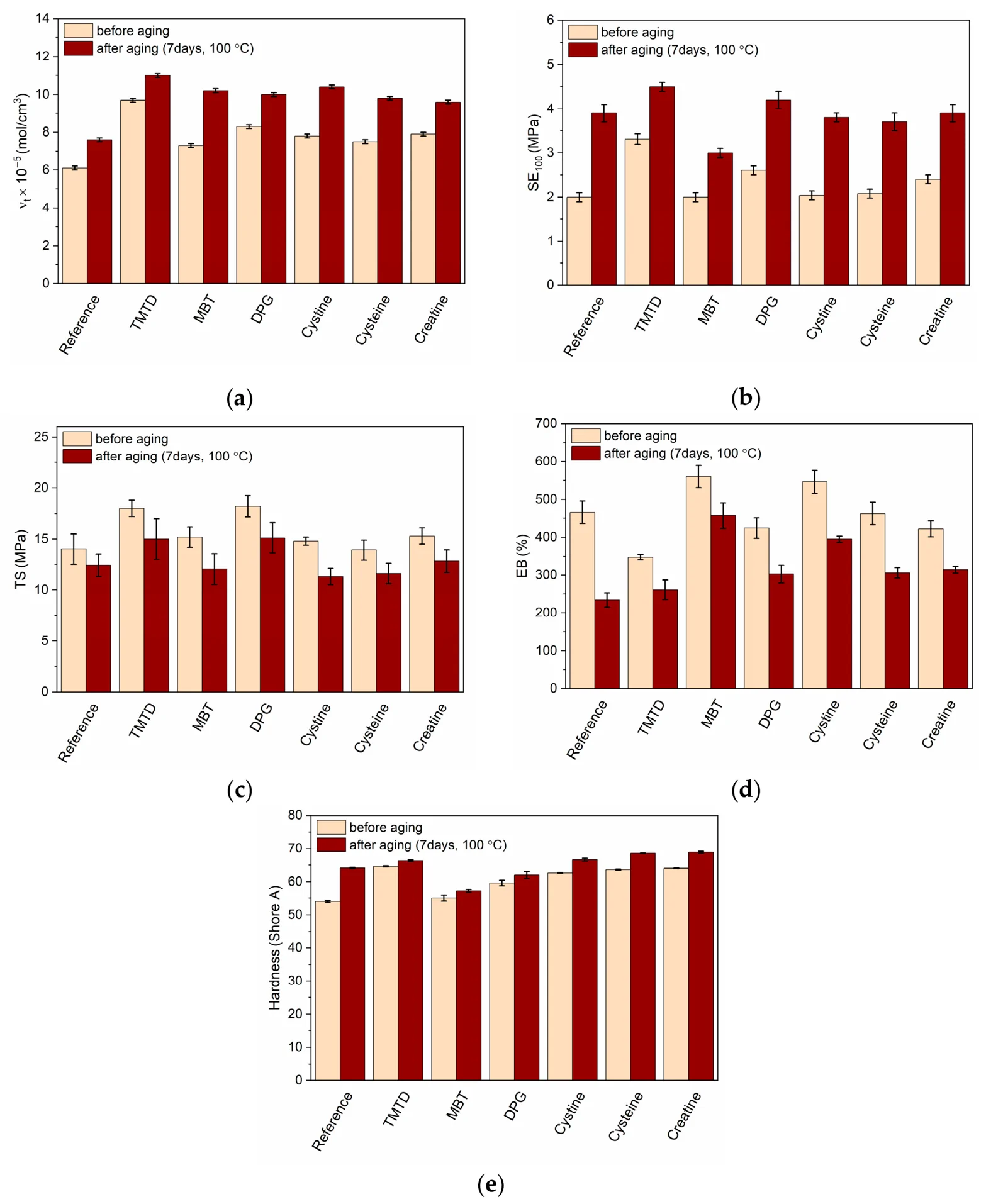
Effect of thermo-oxidative aging on the properties of carbon black-filled NBR vulcanizates: (**a**) crosslink density; (**b**) stress at 100% elongation; (**c**) tensile strength; (**d**) elongation at break; (**e**) hardness.

The service performance of rubber materials depends not only on their mechanical strength and other functional properties but also on their resistance to aging, which determines their long-term stability under thermo-oxidative conditions. This is especially relevant for carbon black-filled elastomer composites, as reinforcing fillers are introduced not only to improve the immediate mechanical properties but also to maintain these properties throughout prolonged service. Therefore, the resistance to thermo-oxidative aging of NBR vulcanizates was investigated, and the results are shown in Figure 5 as the changes in the mechanical properties and crosslink density of the vulcanizates due to aging.

**Figure 6:**
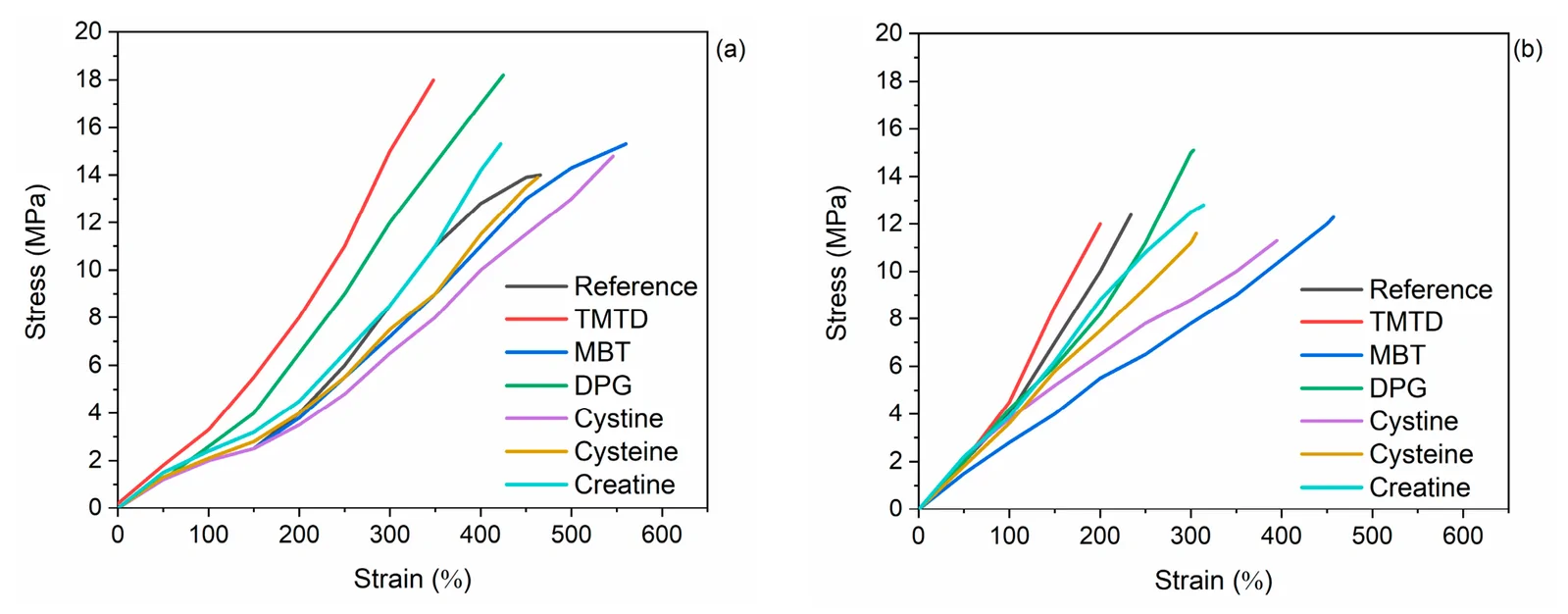
Stress–strain curves of carbon black-filled NBR vulcanizates: (**a**) before aging process; (**b**) after aging.

For a more comprehensive visualization of the mechanical response and the effect of thermo-oxidative aging, stress–strain curves are also presented in Figure 6. They complement the tensile parameters by illustrating the complete deformation behavior and changes in the stiffness and extensibility of the investigated vulcanizates. The plotted curves represent the average tensile response obtained for each formulation.

Thermo-oxidative aging resulted in an increase in the SE_100_ values of all NBR vulcanizates (Figure 5b), indicating enhanced stiffness of the elastomer network, which was consistent with the increase in crosslink density observed after aging. As expected, thermo-oxidative aging promoted further development of the crosslinked network in all investigated vulcanizates (Figure 5a), leading to an increase in the crosslink density as a result of the post-curing phenomenon commonly observed in sulfur-cured elastomers [36,37]. The most pronounced increase in the crosslink density was observed for the vulcanizate with MBT, suggesting that this curing system retained the greatest potential for further network development during thermo-oxidative aging. This was probably due to the lower crosslink density of this vulcanizate before the aging processes. The broad crosslinking peak from DSC analysis also confirmed that the crosslinking process may proceed further during long-term exposure to elevated temperatures. It may also suggest that a greater fraction of reactive species remained available after vulcanization, allowing further network development during thermo-oxidative aging. Moreover, MBT may be partially adsorbed onto the carbon black surface through its benzothiazole moiety (via π–π interactions), reducing its effective concentration during vulcanization [38,39]. As a result, additional crosslinking reactions may continue during aging, contributing to the pronounced increase in crosslink density observed for the MBT-containing vulcanizate. As expected, the increase in crosslink density resulting from thermo-oxidative aging caused a reduction in TS of all investigated vulcanizates (Figure 5c). This behavior is characteristic of sulfur-cured elastomers, in which the increased density of the crosslinked network reduces the ability of polymer chains to orient and distribute the applied stress during tensile deformation. A corresponding decrease in EB was also observed for all vulcanizates (Figure 5d), with the most pronounced reduction occurring for the reference vulcanizate. This finding is consistent with the remaining results, indicating that thermo-oxidative aging promoted the formation of a stiffer and less deformable elastomer network. Consequently, the hardness of all vulcanizates also increased after aging, confirming the progressive stiffening of the materials induced by the post-curing process (Figure 5e).

Compared with our previous studies [12] concerning unfilled NBR vulcanizates, thermo-oxidative aging of the carbon black-filled composites resulted in a decrease rather than an increase in TS, despite the observed increase in crosslink density. This difference may be attributed to the reinforcing effect of carbon black, which provides high initial stiffness and mechanical strength before aging. Consequently, further network development during post-curing no longer improves the tensile properties but instead increases the rigidity of the elastomer network, thereby limiting chain orientation during tensile deformation.

The stress–strain curves presented in Figure 6 showed that before the aging process (Figure 6a), the TMTD- and DPG-containing vulcanizates exhibited the highest stress levels, consistent with their higher crosslink densities and SE_100_ values, whereas vulcanizates containing MBT and cystine exhibited greater deformability. The amino acid-based systems generally displayed intermediate tensile responses, with cystine exhibiting particularly high elongation. After thermo-oxidative aging (Figure 6b), all vulcanizates showed increased stiffness and reduced elongation, as evidenced by the steeper stress–strain curves and lower strains at break. This behavior was consistent with the observed increase in crosslink density and SE_100_ resulting from post-curing, accompanied by reductions in tensile strength and elongation at break. The changes were particularly pronounced for the TMTD-containing vulcanizate, while the systems based on MBT and amino acids generally retained greater deformability after aging.

To more comprehensively evaluate the resistance of the carbon black-filled NBR vulcanizates to thermo-oxidative aging, the aging coefficient (A_f_) was determined. This parameter was calculated based on the changes in TS and EB values before and after the aging process, providing an overall assessment of the retention of useful properties of rubber composites under the aging process. The results are given in Table 5.

The aging coefficient (A_f_) followed a similar trend to that observed in our previous study on unfilled NBR vulcanizates. Among the vulcanizates containing the investigated accelerators, the poorest aging resistance was observed for the TMTD-containing vulcanizate (Table 5), which was characterized by an A_f_ of 0.5, whereas the reference formulation exhibited a lower A_f_ value of 0.4. This finding may result from the higher proportion of thermally unstable polysulfidic crosslinks, which can be formed in the presence of TMTD or in the case of unaccelerated sulfur vulcanization. These crosslinks are prone to thermal scission and rearrangement during aging [40,41]. At the same time, post-curing reactions further increased the crosslink density, producing an overly rigid elastomer network with limited chain mobility. Consequently, the vulcanizate became more brittle, leading to a greater deterioration of its mechanical performance after thermo-oxidative aging. Furthermore, the carbon black-filled NBR vulcanizates containing amino acids exhibited mechanical properties comparable to those of the vulcanizates cured with conventional accelerators. Analysis of the aging coefficient further confirmed that both the conventional and amino acid-based curing systems provided similar resistance to thermo-oxidative aging, with A_f_ values remaining within a relatively narrow range of 0.5–0.6. These findings demonstrate that replacing commercial vulcanization accelerators with amino acids did not compromise either the mechanical performance or the long-term stability of carbon black-filled NBR vulcanizates. Consequently, amino acids may be considered promising, environmentally benign and health-friendly alternatives to conventional vulcanization accelerators, particularly for rubber products, where reducing the use of potentially allergenic or harmful conventional additives is desirable.

### 2.5. Thermal Stability of NBR Vulcanizates

**Figure 7:**
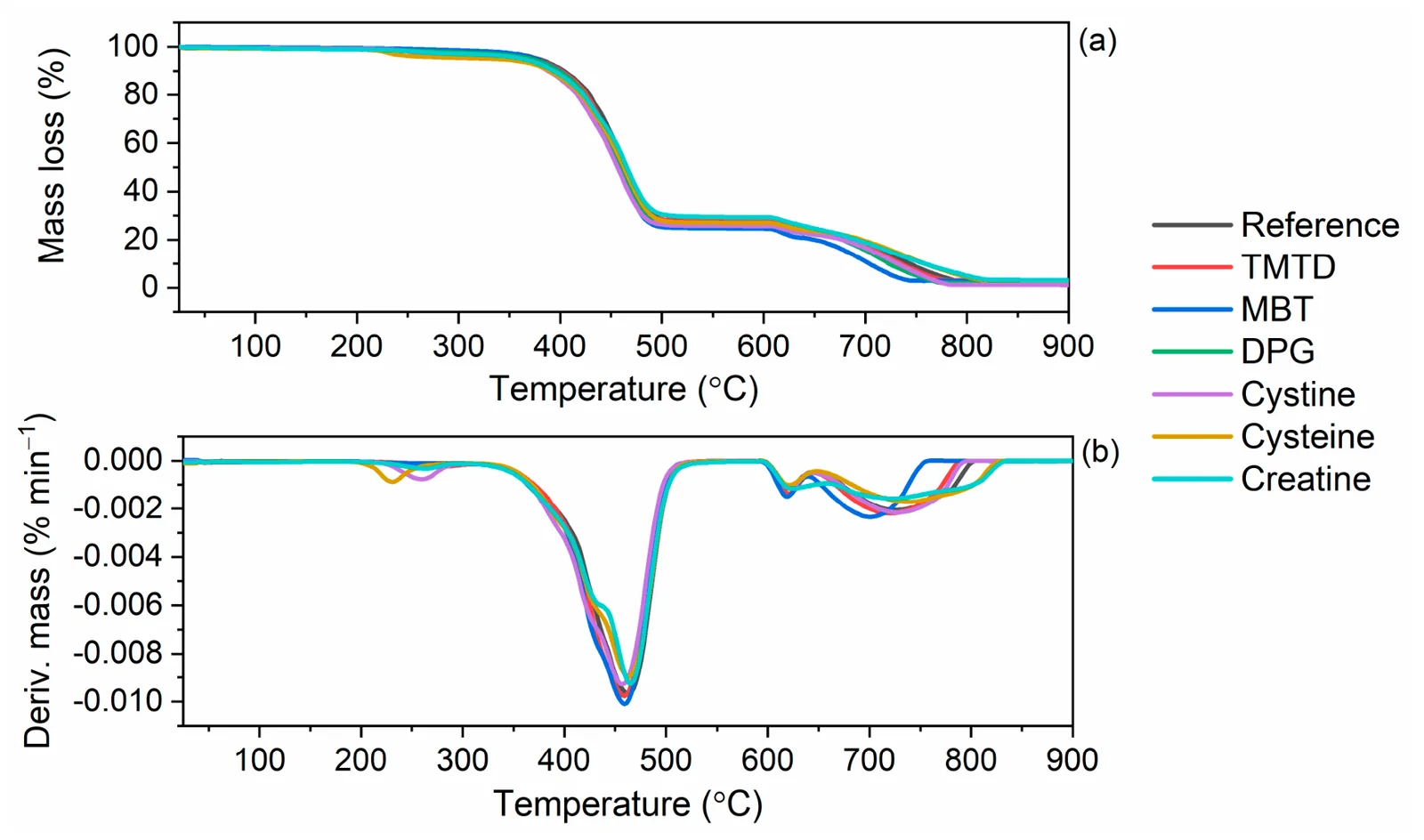
Thermal stability of carbon black-filled NBR vulcanizates: (**a**) TG curves, (**b**) DTG curves.

To further evaluate the influence of amino acids on the performance of carbon black-filled NBR vulcanizates, thermogravimetric analysis (TGA) was performed. This technique provides information on the thermal stability of the crosslinked elastomer network and enables assessment of the influence of both the curing system and the reinforcing filler on the decomposition behavior of the composites. The samples were heated from 25 to 600 °C under argon in order to investigate the thermal decomposition of the elastomer network without oxidative reactions. Subsequently, the atmosphere was switched to air, and the temperature was increased to 900 °C to evaluate the oxidation of the carbonaceous residue and carbon black. The obtained results are summarized in Table 6, whereas the TG and DTG curves are presented in Figure 7.

The thermal degradation of all vulcanizates proceeded in two distinct stages (Figure 7). The first stage, occurring under an inert atmosphere, was associated with the decomposition of the sulfur-crosslinked NBR elastomer matrix, whereas carbon black remained practically unchanged owing to its excellent thermal stability in an inert atmosphere [42]. The second stage corresponded to the oxidative degradation of the carbonaceous residue and the combustion of carbon black after switching the atmosphere from argon to air. Such behavior is characteristic of carbon black-filled elastomeric composites [11,30,32,42,43].

The onset decomposition temperature (T_5%_) depended on the curing system. The reference vulcanizate exhibited the highest value (380 °C), while slightly lower temperatures were obtained for vulcanizates, which contained conventional accelerators (370–375 °C), probably due to the limited thermal stability of accelerators. The incorporation of amino acids resulted in a further decrease in T_5%_, particularly for cystine (347 °C) and cysteine (331 °C), whereas creatine showed intermediate behavior (363 °C). The reduced onset decomposition temperatures observed for sulfur-containing amino acids are most likely associated with the lower thermal stability of their sulfur-containing functional groups, which may undergo decomposition before degradation of the NBR backbone. Similar thermal behavior of cystine and cysteine was observed in our previous study performed on unfilled NBR, where these amino acids also exhibited earlier thermal decomposition than the commercial accelerators owing to cleavage of disulfide or thiol-containing structures [12]. Despite these differences in T_5%_, the temperatures corresponding to the maximum decomposition rate remained remarkably similar for all vulcanizates (467–474 °C). This indicates that the degradation mechanism of the main NBR chains was essentially unaffected by the type of curing system. Therefore, the differences introduced by amino acids mainly influenced the initial stage of thermal degradation, while the thermal stability of the crosslinked elastomer network itself remained comparable regardless of the accelerator employed.

The mass loss recorded between 200 and 600 °C ranged from 70.3 to 75.2%, indicating that decomposition of the organic elastomer phase proceeded similarly for all curing systems. Following the introduction of air, additional mass losses of 22.0–26.2% were observed between 600 and 900 °C. This stage corresponded predominantly to the oxidation of the carbonaceous residue and combustion of carbon black. Considering that the formulations contained 30 phr of carbon black, corresponding to approximately 22–23 wt.% of the total compound, the observed mass losses are consistent with the expected carbon black content. A similar TG/DTG profile has previously been reported for carbon black-filled vulcanizates [30,44,45], further supporting the interpretation of the thermal degradation stages observed in the present study. The relatively similar mass losses obtained for all vulcanizates confirm that the amount of carbon black remained constant in all formulations and that the type of accelerator did not influence its thermal oxidation. Consequently, only a small inorganic residue (1.4–3.6%) remained at 900 °C.

Compared with our previous study on unfilled NBR vulcanizates, an important difference can be observed. In the absence of reinforcing filler, thermal stability was governed primarily by the structure of the sulfur crosslinks and the resulting network architecture. In contrast, for the carbon black-filled composites investigated in the present work, thermal stability depended not only on the curing system but also on the reinforcing effect of carbon black. It should be noted that carbon black may act as a thermal barrier, limiting heat transfer and diffusion of volatile degradation products and stabilizing the carbonaceous residue formed during pyrolysis. As a consequence, although amino acids affected the onset of degradation, they had only a minor influence on the temperature of the main decomposition stage. Thus, the thermal stability of the filled vulcanizates was governed predominantly by the elastomer matrix together with the reinforcing filler rather than by the accelerator itself. This resulted in higher T_5%_ of carbon black-filled vulcanizates compared to the unfilled ones [12].

Most importantly, although the use of amino acids as an alternative to conventional accelerators lowered the temperature at which thermal decomposition begins, the vulcanizates are still stable up to temperatures above 330 °C, so considering the applications of NBR composites, this should not be a significant factor. Moreover, the observed differences were limited mainly to the initial decomposition stage, whereas the principal degradation process of the NBR matrix remained practically unchanged.

## 3. Materials and Methods

### 3.1. Materials

**Figure 8:**
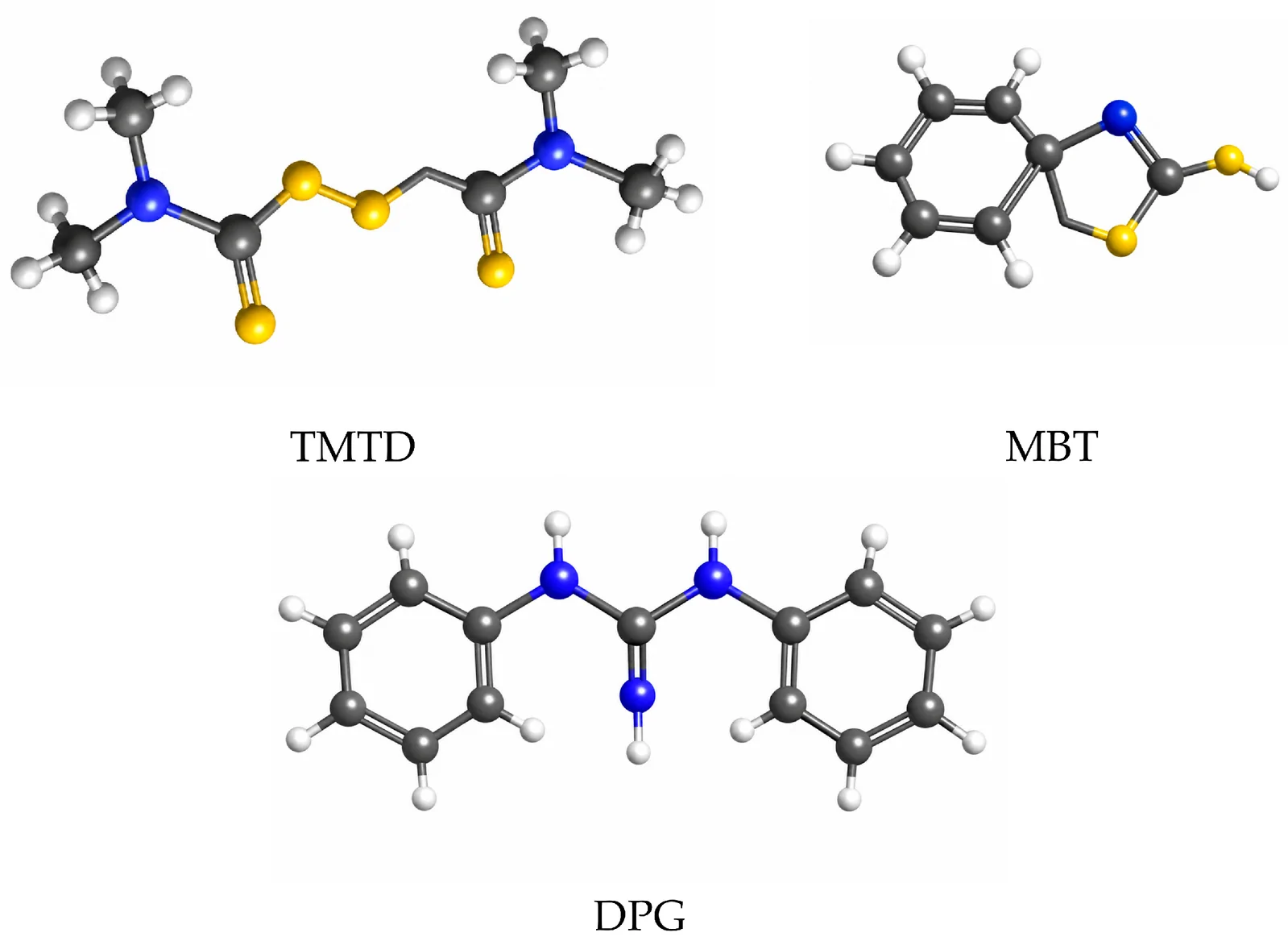
The structure of commercial accelerators.

**Figure 9:**
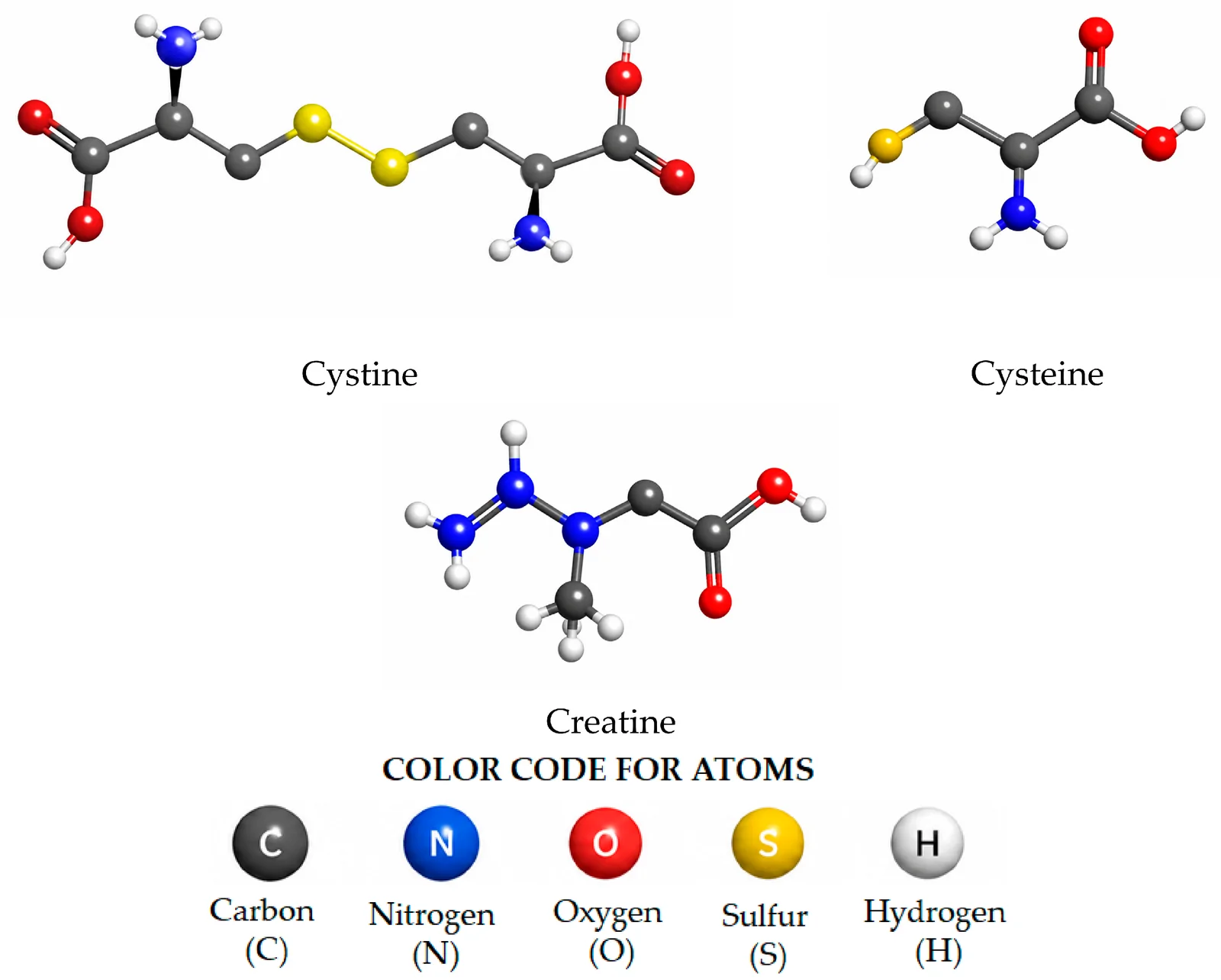
The chemical structures of amino acids.

Acrylonitrile–butadiene rubber (NBR, grade SKN3365E) was used as the elastomer matrix. The rubber contained 31–35 wt.% acrylonitrile and exhibited a Mooney viscosity, ML(1+4) at 100 °C, of 62–68. The NBR was supplied by Konimpex Chemicals (Konin, Poland). The rubber compounds were reinforced with carbon black N550 (Konimpex Chemicals, Konin, Poland), which served as the filler. The carbon black had a specific surface area of 40 m^2^·g^−1^ and a pH ranging from 7 to 10. Reference rubber compounds were prepared using three commercially available sulfur vulcanization accelerators: 2-mercaptobenzothiazole (MBT), 1,3-diphenylguanidine (DPG), and tetramethylthiuram disulfide (TMTD). All accelerators were purchased from Sigma-Aldrich (Schnelldorf, Germany), and their chemical structures are shown in Figure 8. To investigate potential alternatives to conventional accelerators, three amino acids with different chemical structures—L-cystine, L-cysteine, and creatine—were employed instead of the commercial accelerators. Their chemical structures are presented in Figure 9.

### 3.2. Preparation of Rubber Compounds and Testing Procedures

The formulations of the investigated NBR compounds are summarized in Table 7. All rubber compounds were prepared using a laboratory two-roll mill (David Bridge & Co., Rochdale, UK) with roll dimensions of 200 mm in diameter and 450 mm in length. The front roll rotated at 16 rpm, while the roll gap was maintained between 1.5 and 3.0 mm with a friction ratio of 1:1.2. The average roll temperature during mixing was approximately 30 °C. The compounding process was carried out in two stages. In the first stage, a masterbatch containing NBR and carbon black was prepared to ensure uniform filler dispersion. In the second stage, the remaining ingredients of the curing system, including sulfur (S), zinc oxide (ZnO), stearic acid (St.A.), and the selected accelerator, were incorporated into the masterbatch. For comparison purposes, a control compound without a vulcanization accelerator was also prepared. In addition, three reference rubber compounds containing 2 phr of the commercial accelerators, i.e., TMTD, MBT, and DPG, were produced. In rubber technology, vulcanization accelerators such as MBT, DPG, or TMTD are typically incorporated into rubber compounds in amounts 1–2 phr, depending on their specific role in the curing system and the desired vulcanization profile [6,7]. Alternatively to commercial accelerators, the amino acids, such as L-cystine, L-cysteine, and creatine, were used for the preparation of separate rubber formulations. The amino acid loading of 5 phr was selected based on preliminary experiments, in which a lower loading did not provide a satisfactory effect on the vulcanization process. This loading was subsequently adopted in our previous study [12] and was retained in the present work to ensure consistency and enable a direct comparison between the unfilled and carbon black-filled NBR systems. General recipes of the rubber compounds are presented in Table 7.

The vulcanization behavior of the prepared rubber compounds was investigated using a rotorless rheometer (D-RPA 3000, MonTech, Buchen, Germany) at a test temperature of 160 °C, following the requirements of ISO 6502-19 [46]. Three independent measurements were performed for each rubber compound formulation. The rheometric data obtained from the curing curves were used to determine the characteristic curing parameters, including the scorch time (t_5_) and the optimum cure time (t_90_).

Differential scanning calorimetry (DSC1, Mettler Toledo, Greifensee, Switzerland) was employed to investigate the curing reactions in the prepared rubber compounds. The analysis was conducted according to ISO 11357-1:2020 [47]. Samples enclosed in aluminum pans were heated over the temperature range from −150 to 250 °C at a heating rate of 10 °C·min^−1^ in an inert argon atmosphere. The resulting heat-flow curves were used to evaluate the thermal phenomena associated with sulfur vulcanization. For each formulation, a single DSC measurement was performed, and the obtained parameters are reported as individual values. According to the STARe software (v.16.40), the instrumental uncertainty associated with the DSC measurements was ±1 °C for T_g_ and ±2 J g^−1^ for the enthalpy change (ΔH).

The crosslink density of the cured rubber compounds was determined by equilibrium swelling measurements in toluene, performed in accordance with ISO 1817:2021 [48]. For each formulation, the measurements were performed using four independent specimens. The swelling results were analyzed using the Flory–Rehner theory [49], which enables the calculation of the network crosslink density from the equilibrium swelling behavior of the vulcanizates. The crosslink density (ν_t_) was calculated using Equation (1):

(1) $$\nu_{t}=-\left[ln\left(1-V_{r}\right)+V_{r}+\mu \cdot V_{r}^{2}\right]/\left[V_{0}\cdot \left(V_{r}\left(1/3\right)\right)-\left(V_{r}/2\right)\right],$$

where ν_t_ denotes the crosslink density, V_r_ is the volume fraction of rubber in the swollen network, V_0_ represents the molar volume of the swelling solvent (106.27 cm^3^·mol^−1^ for toluene), and μ is the polymer–solvent interaction parameter [50]. The value of μ was determined from Equation (2) [12,51], which is the Huggins parameter and defines the interaction between elastomer and solvent:

(2) $$µ=0.3809+0.6707V_{r},$$

The effect of amino acids on the thermal stability of carbon black-filled NBR vulcanizates was evaluated by thermogravimetric analysis (TGA) using a TGA/DSC1 analyzer (Mettler Toledo, Greifensee, Switzerland). The measurements were carried out over the temperature range of 25–900 °C at a constant heating rate of 10 °C·min^−1^. The samples were heated under an argon atmosphere (50 mL·min^−1^) up to 600 °C, after which the purge gas was switched to air and heating was continued to the final temperature. For each formulation, TGA was performed using a single measurement. Therefore, the results are reported as individual values. The instrumental uncertainty reported by the STARe software was ±1.0 °C for the temperature of the beginning of thermal decomposition (T_5_%), ±1.0 °C for DTG peak temperature (T_DTG_), and ±1.0% for mass loss resulting from decomposition (Δm).

The dispersion of carbon black throughout the NBR elastomer matrix was characterized by scanning electron microscopy (SEM). Microstructural observations were carried out using a LEO 1450 scanning electron microscope (Carl Zeiss AG, Oberkochen, Germany). Prior to imaging, the vulcanized specimens were cryogenically fractured after immersion in liquid nitrogen to obtain fresh fracture surfaces. To ensure sufficient electrical conductivity during SEM analysis, the fractured surfaces were coated with a thin gold layer before examination.

Mechanical performance under tensile loading was characterized according to the ISO 37 standard [52] using a Zwick/Roell Z1435 universal testing machine (Ulm, Germany). Dumbbell specimens, approximately 2 mm thick, were stretched at a constant crosshead speed of 500 mm·min^−1^ until failure. For each vulcanizate, five independent dumbbell specimens were tested.

The hardness of the vulcanized NBR compounds was measured on disk-shaped specimens using the Shore A scale. Measurements were performed with a Zwick/Roell 3105 digital durometer (Ulm, Germany) in accordance with the requirements of ISO 868 [53]. For each specimen, six independent hardness measurements were performed at different locations, each at least 5 mm from the specimen edge.

The viscoelastic behavior of the vulcanizates was characterized by dynamic mechanical analysis (DMA) [54] using a DMA/SDTA861e instrument (Mettler Toledo, Greifensee, Switzerland) operated in tensile mode. During testing, the specimens were subjected to sinusoidal deformation with an oscillation amplitude of 4 μm at a frequency of 1 Hz. The storage modulus (E′) and loss modulus (E″) were continuously recorded while heating the samples from −100 to 70 °C at a constant rate of 3 °C·min^−1^. Two independent specimens were tested for each formulation.

The thermo-oxidative stability of the NBR vulcanizates was investigated in accordance with the ISO 188 standard [55]. Vulcanized sheets with a thickness of approximately 2 mm were subjected to accelerated thermo-oxidative aging in a laboratory drying oven (Binder, Tuttlingen, Germany) at 100 °C for 168 h (7 days). After the aging process, the changes in crosslink density and hardness were determined and compared with the corresponding values obtained for the unaged samples to evaluate the extent of material degradation. The properties after aging were evaluated using the same number of specimens and measurement procedures as those applied before aging. Following the thermo-oxidative aging procedure, NBR vulcanizates were evaluated to determine the changes in their mechanical properties induced by aging. Based on the tensile strength and elongation at break measured before and after aging, the aging coefficient (A_f_) was calculated according to Equation (3) [56,57].

(3) $$A_{f}={\left(TS\times EB\right)}_{after\;aging\;}/{\left(TS\times EB\right)}_{before\;aging},$$

For all investigated properties, the results obtained from the independent measurements were averaged for each formulation and are presented and interpreted as mean ± standard deviation (SD). The influence of both the accelerator type (conventional versus amino acid-based) and carbon black filler on the crosslink density, vulcanization behavior, thermal stability, and mechanical strength of the elastomer composites was verified using one-way ANOVA. This statistical tool allowed for a rigorous comparison of mean values between the formulated groups. Differences were considered statistically significant when the *p*-value was below 0.05, representing a 95% confidence level. The conducted analysis verified that the choice of curing accelerator type and the incorporation of carbon black filler had a statistically significant impact on the final characteristics of the elastomer composites (*p* < 0.05).

## 4. Conclusions

The present study demonstrated that naturally occurring amino acids, namely L-cystine, L-cysteine, and creatine, remain active sulfur vulcanization accelerators in carbon black-filled NBR compounds despite the additional filler–curative interactions introduced by the reinforcing carbon black. Although their curing activity was lower than that of conventional commercial accelerators, all investigated amino acids promoted sulfur crosslinking compared to unaccelerated sulfur vulcanization, leading to well-developed elastomer networks with crosslink densities, thermal stability, dynamic mechanical behavior, and mechanical performance comparable to those of conventionally accelerated vulcanizates. Among the investigated bio-based curing systems, creatine exhibited the highest accelerating activity, whereas cystine provided the most favorable overall balance between curing characteristics, crosslinking efficiency, thermal behavior, mechanical performance, and thermo-oxidative aging resistance. The differences in the properties of the vulcanizates are likely related mainly to the curing mechanism and the resulting elastomer network structure, although the contribution of carbon black dispersion cannot be excluded.

Compared with our previous study on unfilled NBR, the present work demonstrates that the beneficial effects of amino acid-based curing systems are preserved in reinforced rubber compounds, although the presence of carbon black modifies the curing behavior through filler–curative interactions. At the same time, the reinforcing effect of carbon black becomes an additional factor governing the final mechanical performance and thermo-oxidative aging behavior of the vulcanizates. These findings indicate that the activity of amino acids is retained under technologically relevant conditions representative of rubber formulations.

Overall, the results indicate that natural bio-additives, such as amino acids, represent a promising alternative to conventional, potentially allergenic vulcanization accelerators. For NBR products designed for prolonged skin contact, utilizing these additives could address the growing demand to minimize exposure to hazardous accelerator residues, which are commonly linked to allergic contact dermatitis, despite not achieving the shortest possible curing time. Their promising performance in carbon black-filled formulations, despite slower curing kinetics, supports further investigation of amino acid-based systems as potential alternatives to conventional allergenic vulcanization accelerators and may advance their applicability in healthcare elastomer products.

## Figures and Tables

**Table 1:** Cure parameters at 160 °C of carbon black-filled NBR compounds.

Accelerator	S_min_ (dNm)	∆S (dNm)	t_5_ (min)	t_90_ (min)
Reference	1.3	11.7	9	95
TMTD	1.1	15.2	1	9
MBT	1.2	9.3	1	30
DPG	1.2	12.6	1	39
Cystine	1.2	12.0	10	81
Cysteine	1.3	11.0	11	78
Creatine	1.3	13.1	10	70

S_min_, minimum torque during rheometric measurement; ∆S, torque increment; t_5_, scorch time; t_90_, optimal vulcanization time; SD: S_min_ ± 0.1 dNm, ΔS ± 0.2 dNm; t_5_ ± 0.1 min; t_90_ ± 0.3 min.

**Table 2:** Crosslinking temperature and enthalpy of carbon black-filled NBR compounds determined by differential scanning calorimetry (DSC).

Accelerator	T_g_ (°C)	T_cross_ (°C)	∆H (J/g)
Reference	−28	192-247	11.5
TMTD	−27	163-173	14.6
MBT	−27	156-185	4.6
DPG	−28	177-221	4.3
Cystine	−28	172-243	16.8
Cysteine	−28	179-221	7.8
Creatine	−28	175-243	14.8

T_g_, glass transition temperature; T_cross_, temperature of crosslinking; ∆H, enthalpy of crosslinking.

**Table 3:** Tensile properties and hardness of carbon black-filled NBR vulcanizates.

Accelerator	SE_100_ (MPa)	TS (MPa)	EB (%)	H (Shore A)
Reference	2.0 ± 0.1	14.0 ± 0.2	466 ± 30	54 ± 1
TMTD	3.3 ± 0.1	18.0 ± 0.1	348 ± 7	65 ± 1
MBT	2.0 ± 0.1	15.2 ± 0.2	560 ± 29	55 ± 1
DPG	2.6 ± 0.1	18.2 ± 0.2	424 ± 27	60 ± 1
Cystine	2.0 ± 0.1	14.8 ± 0.1	546 ± 30	63 ± 1
Cysteine	2.1 ± 0.1	13.9 ± 0.1	463 ± 30	64 ± 1
Creatine	2.4 ± 0.1	15.3 ± 0.1	422 ± 21	64 ± 1

SE_100_, stress at relative elongation of 100%; TS, tensile strength; EB, elongation at break; H, hardness.

**Table 4:** Glass transition temperature (T_g_) and mechanical loss factor (tan δ) of NBR vulcanizates determined by dynamic mechanical analysis (DMA).

Accelerator	T_g_ (°C)	tan δ_Tg_ (−)	tan δ_25 °C_ (−)	tan δ_60 °C_ (−)
Reference	−19 ± 1	1.4 ± 0.1	0.14 ± 0.02	0.08 ± 0.01
TMTD	−15 ± 1	1.5 ± 0.1	0.11 ± 0.02	0.05 ± 0.01
MBT	−20 ± 1	1.5 ± 0.1	0.14 ± 0.02	0.09 ± 0.01
DPG	−18 ± 1	1.4 ± 0.1	0.11 ± 0.02	0.07 ± 0.01
Cystine	−21 ± 1	1.3 ± 0.1	0.14 ± 0.02	0.13 ± 0.01
Cysteine	−21 ± 1	1.3 ± 0.1	0.15 ± 0.02	0.13 ± 0.01
Creatine	−20 ± 1	1.3 ± 0.1	0.14 ± 0.02	0.12 ± 0.01

**Table 5:** Thermo-oxidative aging factor (A_f_) of carbon black-filled NBR vulcanizates.

Accelerator	A_f_ (−)
Reference	0.4 ± 0.1
TMTD	0.5 ± 0.1
MBT	0.6 ± 0.1
DPG	0.6 ± 0.1
Cystine	0.5 ± 0.1
Cysteine	0.6 ± 0.1
Creatine	0.6 ± 0.1

**Table 6:** Thermal stability of carbon black-filled NBR vulcanizates evaluated by thermogravimetric analysis (TGA).

Accelerator	T_5%_ (°C)	T_DTG_ (°C)	∆m_200–600 °C_ (%)	∆m_600–900 °C_ (%)	Residue at 900 °C (%)
Reference	380	467	71.7	25.0	3.3
TMTD	373	471	71.5	25.0	3.5
MBT	375	473	75.2	22.0	2.8
DPG	370	469	72.8	25.0	2.2
Cystine	347	470	73.9	24.7	1.4
Cysteine	331	467	72.3	24.1	3.6
Creatine	363	474	70.3	26.2	3.5

T_5%_, temperature of the beginning of thermal decomposition; T_DTG_, DTG peak temperature; ∆m, mass loss resulting from decomposition.

**Table 7:** General recipes of the NBR compounds filled with carbon black, phr (parts per hundred of rubber).

Compounds	Reference	TMTD	MBT	DPG	Cystine	Cysteine	Creatine
Reference	100	100	100	100	100	100	100
S	2	2	2	2	2	2	2
ZnO	5	5	5	5	5	5	5
St.A.	1	1	1	1	1	1	1
CB	30	30	30	30	30	30	30
TMTD	-	2	-	-	-	-	-
MBT	-	-	2	-	-	-	-
DPG	-	-	-	2	-	-	-
Cystine	-	-	-	-	5	-	-
Cysteine	-	-	-	-	-	5	-
Creatine	-	-	-	-	-	-	5

## Data Availability

The raw data supporting the conclusions of this article will be made available by the authors on request.
